# Sharing intention of electronic health records in online health communities: Patients’ behavioral decisions in the context of privacy protection measures

**DOI:** 10.3389/fpsyg.2022.1047980

**Published:** 2022-12-22

**Authors:** Shanshan Guo, Yuanyuan Dang, Bofei She, Yugang Li

**Affiliations:** ^1^School of Business and Management, Shanghai International Studies University, Shanghai, China; ^2^School of Business Administration, South China University of Technology, Guangzhou, China; ^3^School of Management, Harbin Institute of Technology, Harbin, Heilongjiang, China

**Keywords:** electronic health records sharing, online health community, health information sharing intention, privacy protection, health information

## Abstract

Online health communities (OHCs) have become more important to people’s daily lives on the foundation of the voluntary sharing of electronic health records (EHRs). However, no in-depth investigation has been conducted concerning the influence of the perceptions of privacy protection among patients on their willingness to share EHRs. To fill the knowledge gap, by combining and modifying the theory of planned behavior (TPB) and the health belief model in the context of the privacy protection models implemented by OHCs, an empirical research method using a questionnaire approach is conducted to validate the hypotheses. The results indicate that the more positive a patient’s attitude toward medical information sharing behavior is, the higher that patient’s level of perceived behavioral control; in addition, the greater the social rewards obtained from this process, the more willing the patient is to share his or her EHRs after privacy protection measures are implemented by OHCs. Meanwhile, the effects of past positive experiences and disease severity have also been tested. The findings of this study can be used to promote patients’ full participation in OHCs from a privacy perspective and offer theoretical and practical suggestions to promote the development of OHCs.

## 1. Introduction

### 1.1. Electronic health records shared in online health communities

Due to the continuous development of health information technology, the emergence of online health communities (OHCs) has greatly facilitated interactions between physicians and patients (Cotten and Gupta, 2004; Jadad et al., 2006; Guo et al., 2017, 2018). These interactions are based on sharing and exchanging personal electronic health records (EHRs) on the part of patients through the use of OHCs. EHRs are defined in terms of all information pertaining to the following factors related to the patient or their family: physical or mental health, medical history, treatment process, treatment outcomes and satisfaction, medications taken, experiences with medical care or treatment, and other health-related information posted by the patient as part of his or her participation in the community (Escoffery et al., 2008). For example, a patient must disclose information regarding his or her health condition, illnesses, symptoms, and medications to the doctor or other patients when consulting a doctor, and when sharing experiences of previous medical care, the related treatment methods, therapeutic effects, etc., should also be shared. As a platform for information sharing and interaction between doctors and patients or between patients and other patients, the personal EHR shared by patients in the context of OHCs can help doctors working online test the effectiveness of their diagnoses and treatments and improve their treatment methods, which can also alleviate the difficulties caused by information asymmetry and a shortage of medical resources. On the other hand, an EHR shared with other patients (such as PatientLikeMe) can enrich the health knowledge available in OHCs and serve as a reference for other patients, which can promote the level of health care available to other patients (Dang et al., 2020). Therefore, the voluntary sharing of personal EHRs is the foundation of the development of online health communities.

### 1.2. Privacy protection measures in online health communities

To facilitate EHR sharing behavior in the context of OHCs, studies have explored ways of allowing OHCs to protect EHRs shared by patients in legal or technical terms (Scheibner et al., 2021). As incidents of data leakage increase, the sharing behavior of users of a platform is influenced by the patient privacy protection policies implemented by the platform (Rainie and Duggan, 2016). Recent studies have shown that only a small percentage of users trust the personal data protection services of such platforms (Aïmeur et al., 2016). The key barriers to EHR sharing acceptance are health care coverage, privacy, and the security of EHRs. When users gradually discover that platforms generate revenue from the data that users share online, this discovery contradicts the patients’ desire to protecting their personal privacy, which can influence users’ beliefs regarding the platform and further influence their willingness to share EHRs *via* the platform. In fact, OHCs have gradually implemented a series of privacy protection measures, such as concealing patients’ personal information (e.g., name/gender/address/contact information), ensuring that test results such as images are visible only to doctors, and intelligently identifying the privacy of offline treatment information (hospital/section/doctor visited, etc.). In other words, in existing OHC applications, the EHRs shared by patients are already the target of some privacy protection strategies to offset the privacy concerns expressed by some patients and to increase patients’ willingness to share their EHRs. However, some studies have noted that most patients have no clear and specific understanding of health care privacy (McGuire et al., 1985). Studies have shown that most internet users do not read lengthy privacy policies in their entirety when sharing information (Furnell and Phippen, 2012; Ermakova et al., 2014). In the context of sensitive health information, people merely tend to make intuitive judgments regarding whether to disclose such information and do not give much attention to privacy leaks or levels of protection (Yawn et al., 1998; Merz et al., 1999). Additionally, if patients desire to use an OHC platform, they must agree to the platform’s privacy policy, or they may be unable to use the services offered the platform in full. However Patients are unable to choose which parts of their information they desire to disclose when they agree to the current privacy policy (Kim et al., 2019).

In summary, most existing studies have focused on the protection methods used with respect to patients’ shared EHRs in OHCs (i.e., in terms of network technology, sharing protocol, or hardware services) but have not explored the impact of users’ perceptions of the privacy protection policy used for patients in OHCs on those patients’ willingness to share. Therefore, the effectiveness of the privacy protection policies of OHCs (i.e., the ways in which patients make decisions regarding the sharing of EHRs due to their perceptions of the patient privacy protection offered by OHCs) remains unclear and is worth investigating.

### 1.3. Research objective

Privacy calculus theory, which is based on the assumption of rationality, has been widely used in research concerning the protection of health information privacy in the context of OHCs (Dang et al., 2021). Privacy calculus theory explains patients’ online privacy disclosure behaviors in terms of risks and benefits. If the benefits outweigh the risks, patients may choose to disclose their private information (Culnan and Armstrong, 1999), and patients’ access to social support is one of the most important benefits in this context (Dang et al., 2021). That is, patients’ EHR sharing behaviors, such as by sharing their medical history, medications taken, and medical experiences, are not driven by financial gain but rather by the moral needs to help other patients and obtain information and emotional support. However, other studies have noted that people are not fully compliant with the reciprocal privacy calculus in the context of privacy breaches; on the one hand, people exhibit a stronger tendency to refuse to share when their privacy feels threatened, but they occasionally exchange their privacy for small favors (e.g., small gifts) (Dang et al., 2021). On the other hand, patients have different levels of willingness to share different forms of private health care information. With respect to some information, patients prefer to share with medical personnel rather than family members or third-party machines, while with regard to other contexts, such as data from genetic test reports, patients are more likely to share information within their families (Tessaro et al., 1997; Applebaum-Shapiro et al., 2001). Thus, in practice, patients’ decisions concerning EHR sharing are also influenced by psychological factors and personal preferences, which are not entirely identical with rational privacy calculations. In particular, the uncertainty associated with EHR sharing decisions is increased when patients feel that their privacy is protected by OHCs. Therefore, the theory of planned behavior (TPB), which adds “perceived behavioral control” to the theory of rational behavior, is more applicable in this context. Therefore, this study uses an empirical method to investigate the willingness of patients to share their EHRs in OHCs in the context of the privacy protection strategies employed by such platforms. The results of this study can help encourage patients to share EHRs proactively and thus develop the necessary conditions to ensure the sustainable development of OHCs.

### 1.4. Theoretical model construction and hypothesis formulation

#### 1.4.1. Theory of planned behavior

**Figure 1:**
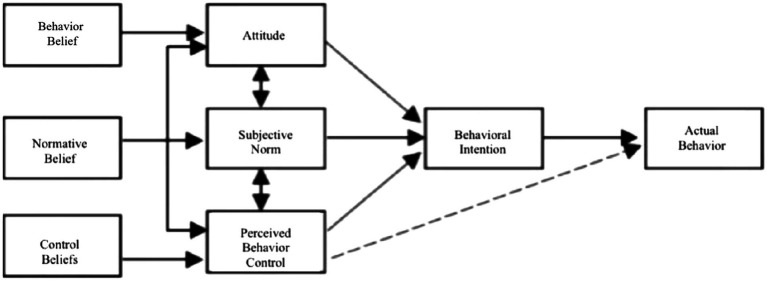
Theory of planned behavior.

The theory of planned behavior (TPB) proposed by Ajzen and Fishbein is the successor to the theory of reasoned action (TRA; Fishbein & Ajzen, 1975). Icek Ajzen found that human behavior is not completely voluntary but rather faces certain forms of control (Ajzen, 1991). Therefore, he added the new concept of “perceived behavior control” to the theory of reasoned action and thus proposed the theory of planned behavior. According to the theory of planned behavior, an individual’s actual action is determined by that individual’s behavioral intention, and this behavioral intention is influenced by the individual’s attitude regarding the behavior, subjective norms and perceived behavioral control. A schematic representation of the theory of planned behavior is shown in Figure 1.

The theory of planned behavior has good explanatory and predictive power with respect to behavior and has been widely used in studies of various forms of behavior, such as consumer behavior, entertainment online behavior, and exercise and learning behavior (Webb and Sheeran, 2006). The theory of planned behavior has been applied in many cases to explain users’ online behavior. For example, Lee used an extended theory of planned behavior to investigate the online transaction behavior of internet users, in which context they added two variables, i.e., trust and perceived risk, to the model and achieved good explanatory effects (Lee, 2009). Some scholars have also applied the theory of planned behavior alongside theories such as those pertaining to privacy concerns to study the information disclosure behavior of users of social networks (Li et al., 2020). In the field of health, the theory of planned behavior has also been used widely. Achterberg et al. combined the theory of planned behavior with other theories to explain people’s dietary and nutritional behaviors (Achterberg and Miller, 2004). The theory of planned behavior can also explain the factors that motivate or hinder people’s actions in order to explain and predict people’s behavior, which can also be used to facilitate interventions in people’s behavior.

Although the theory of planned behavior has been supported and validated by a large number of studies, it has also been revealed to have many shortcomings and deficiencies in the context of long-term empirical tests. In contrast to other affective processing models, the theory of planned behavior has ignored the negative or positive emotions of individuals to some degree (Dutta-Bergman, 2005). Especially in the context of studies concerning health behaviors, emotional feelings are an important variable that cannot be ignored. Therefore, a number of scholars have argued that the existing variables included in the theory of planned behavior cannot explain the behaviors and intentions of individuals adequately. These scholars have tried to add variables such as personality, behavioral experience, and anticipated regret to the model to improve its explanatory power (Sutton, 1994; Aarts et al., 1998). The theory of planned behavior has also been expanded in many respects *via* the addition of a variety of variables. All of these attempts are valuable as long as they lead to an improved explanation of the research problem.

#### 1.4.2. Factors related to planned behavior and willingness to share

According to the theory of planned behavior, actual behavior is determined by behavioral intentions, which are influenced by attitudes, subjective norms, and perceived behavioral control (Ajzen, 1991). Among these factors, subjective norms reflect the behavior beliefs affected by social pressure; however, Sheeran and Orbell argued that social pressure is difficult to investigate directly due to the fact that it is affected by the subjective willingness of others, which indicates that subjective norms cannot effectively reflect the influence of social pressure on individual behavior (Sheeran and Orbell, 1999). Meanwhile, Eisenberger defined social reward in terms of the pleasure, satisfaction, and fulfilment that individuals obtain from interpersonal interactions (Eisenberger et al., 1990). Jiang et al. extended the concept of social rewards and applied it to the online social environment to elaborate its perceived benefits based on privacy calculus theory (Jiang et al., 2013). When individuals realize that a certain social relationship can allow them to receive social rewards, they tend to make greater efforts to maintain or deepen the relationship in question (Schimel et al., 2001). Accordingly, social rewards can reflect the amount of online social pressure to some extent. Previous studies have already used social rewards to explain and predict the online information publishing and sharing behaviors of internet users (Tidwell and Walther, 2002; Jiang et al., 2013). This study focuses on the online environment of OHCs, in which context patient use and sharing behaviors are rarely influenced by their offline social environment but rather depend on their online interactive experiences and the rewards received. Against this backdrop, this study uses social rewards as a replacement for subjective norms to reflect social pressure more adequately. Therefore, we propose that the factors that influence patients’ willingness to share EHRs are social rewards, attitudes, and perceived behavioral control.

#### 1.4.3. The relationship between social rewards and willingness to share

During social interactions, individuals are restricted by the needs for reciprocal exchange and fair information exchange (Lawler and Thye, 1999). Tidwell and Walther studied personal information exchange in the context of computer-mediated communication and found that individuals tend to disclose their personal beliefs, needs, values, etc. (Tidwell and Walther, 2002). to those with whom they share a social interest. In fact, when engaging in social exchange, individuals are expected to share personal information to reciprocate for the benefits provided by others (Lawler and Thye, 1999). In addition, Jiang et al. noted that individuals on the internet are more willing to share their personal information to obtain social rewards and receive personal benefits (Jiang et al., 2013).

First, patients can engage in doctor–patient communication in OHCs to evaluate the effects of treatment and the service attitudes of doctors, share information regarding how to choose doctors, and exchange medical experiences, among other goals. During these processes, patients can help other patients either directly or indirectly and can receive gratitude or respect from those others; meanwhile, they can also obtain psychological satisfaction from helping others. In addition, friendship among patients can be promoted and better interpersonal relationships can be constructed when patients post in OHCs, communicate and share EHRs candidly with doctors and patients online. Previous studies have shown that patients with mental health issues tend to utilize online depression communities to share experiences and exchange valuable information and emotional support to cope with their own diseases (Lu et al., 2021). Moreover, another study verified that the information provided *via* OHCs is moderately understandable and reliable, which can encourage and motivate patients to participate in such communities to improve their health (Kavosi et al., 2020). Specifically, both the psychological satisfaction and the doctor–patient relationship discussed above can be identified as “social rewards.” The existence of social rewards facilitates the exchange and circulation of EHRs in OHCs. If patients who use OHCs are not rewarded for contributing their EHRs, they tend to lose motivation to contribute content in the future. In contrast, when such patients realize the rewards of sharing EHRs voluntarily under conditions characterized by the privacy protection measures of OHCs, they may be more motivated to do so and thus become more active with respect to the subsequent instance of sharing. Therefore, we propose that the more social rewards are available, the more willing patients in OHCs are to share.

*H1*: Social rewards have a positive effect on the intention to share EHRs.

#### 1.4.4. The relationship between attitude and willingness to share

Attitude is a positive or negative feeling experienced by an individual when performing a particular behavior, and this factor has been used widely in behavioral research models. In the target risk assessment (TRA) model, the theory of planned behavior (TPB) model and the technology acceptance model (TAM), attitude has been proven to be directly related to users’ behavioral intentions.

In OHCs, patients must communicate with doctors and other patients to receive medical assistance, engage in experience sharing, etc. Presently, sharing is profitable, and patients can share EHRs pertaining to factors such as their current health conditions or the names and symptoms of their diseases to receive advice concerning how to treat their diseases or study the experiences of other patients with the same conditions. They can also share their experiences with other patients and evaluate the effectiveness of the treatments and services provided by their doctors to gain recognition and appreciation from other patients. However, potential risks are associated with sharing EHRs in the context of personal privacy. Patients in OHCs may have certain privacy concerns when sharing EHRs and may even cease sharing when they encounter unknown risks. Therefore, sharing EHRs can have both positive and negative effects, and patients may intuitively feel positively or negatively regarding such behavior. Specifically, when patients are informed of the privacy protection measures implemented by OHCs, they are willing to share their EHRs if they believe that OHCs can protect them from privacy invasions resulting from their EHR sharing behavior. Conversely, if patients believe that such behavior would be detrimental to them or have a negative impact on them, they tend to be unwilling to share. Pouyan showed that when patients perceive the privacy protection measures implemented by OHCs to be transparent, this perception tends to influence patients’ attitudes and beliefs and ultimately promote patients’ willingness to disclose their health information (Esmaeilzadeh, 2019). Therefore, patients’ positive or negative evaluation of EHR sharing behavior in the context of OHCs’ privacy protection measures, i.e., their attitudes toward such behavior, affect the willingness associated with their behavioral intentions directly.

*H2*: Attitude has a positive effect on intention to share EHRs.

#### 1.4.5. The relationship between perceived behavioral control and willingness to share

Like attitudes, perceived behavioral control has also been widely applied to behavioral research models; this term refers to the individual’s perceived degree of control or mastery when performing a specific behavior. Perceived behavioral control reflects the individual’s past experiences, the availability of second-hand information, and the expected impediments to the behavior. In the TRA, TPB, and TAM models, perceived behavioral control has been shown to have a direct relationship with an individual’s behavioral intention.

Because EHRs have differences from other forms of information, they are associated with a high degree of sensitivity and may be private or intimate. Many uncertainties may arise when patients share EHRs in OHCs, such as concerns that the website may disclose such EHRs to others or that unscrupulous people may steal the individual’s health information. There are also many unknown risks in this context, for example, the possibility that social discrimination may result from privacy leaks. Therefore, the consequences of sharing EHRs are beyond patients’ ability to control. However, patients are willing to share their EHRs in the OHC if they feel that the privacy protections provided by the OHC are effective and they have sufficient information (e.g., concerning privacy settings on the website or legal requirements) to ensure that the information that they post is secure. Additionally, patients are glad to use OHCs to obtain information or help if they believe that they have sufficient ability and the necessary means to address the adverse consequences of posting information. Otherwise, patients are more likely to hesitate or even cease disclosing their EHRs. Therefore, it can be argued that perceived behavioral control directly affects patients’ willingness to share EHRs in OHCs.

*H3*: Perceived behavioral control has a positive effect on the intention to share EHRs.

#### 1.4.6. Health beliefs as salient beliefs

Individuals possess a large number of behavioral beliefs, and the theory of planned behavior identifies salient beliefs as the cognitive and emotional basis for behavioral attitudes, perceived behavioral control and subjective norms. The health belief model was the first theoretical model to explain individual health behaviors; this model was first proposed by the American psychologist Hochbaum in the 1950s and subsequently revised by Becker and Maiman (Wang et al., 2022). The health belief model explains the factors that influence people’s engagement in certain health behaviors from the perspective of the formation of health beliefs (Poss, 2001). The health belief model was first applied to the task of screening people for disease prevention, such as by predicting breast screening behaviors (Yarbrough and Braden, 2001; Wu and Yu, 2003). The health belief model has now been extended to other areas, such as daily exercise behaviors, the prevention of chronic diseases, and intervention in cases of maladaptive behaviors (Hossain et al., 2021). Many studies have confirmed that when explaining and predicting health-related behaviors, the health belief model may function better when combined with other theories of behavior. Some scholars have combined the health belief model with the theory of rational behavior to explain tuberculosis screening behavior (Poss, 2001). Sun, X. sleet et al. suggested that the concepts and connotations of some variables included in the health belief model and the theory of planned behavior are similar and complementary; thus, they can be combined to explain and predict health-related behaviors to increase explanatory power (Sun et al., 2009).

Considering the characteristics of OHCs, we organized the key factors of health beliefs (Glanz et al., 2008) into three factors: disease severity, past positive experiences and EHR sensitivity. First, disease severity is a concept representing an extension of perceived susceptibility and perceived seriousness. Patients’ psychological characteristics and their concerns regarding and perceptions of illness vary across diseases, and health-related behaviors are influenced by the type of illness in question. Some studies have confirmed that people with different disease types exhibit different attitudes and levels of willingness to engage in medical behaviors. Second, past positive experience is a concept that ranges from cue to action. Previous use experiences represent cues regarding the patient’s ability to master actions and can help guide future actions. Positive interaction experiences can promote trust and motivate users’ behaviors (Song, 2007; Bansal and Gefen, 2010). Finally, health information sensitivity is a concept that encompasses the perceived benefits of taking action and the perceived barriers to taking action. Health information sensitivity refers to the degree to which a patient weighs his or her beliefs regarding the barriers to and benefits of shared action at the level of health information privacy protection. Thus, based on the health belief model, we identified the antecedents of the planned action theory as past positive experiences, disease severity, and health information sensitivity.

#### 1.4.7. Influence of past positive experiences on attitudes, social rewards, and perceived behavioral control

Past positive experiences refer to positive experiences that users have encountered during their previous use of OHCs. Previous studies have found that internet users’ past positive experiences on certain websites can increase users’ trust as well as their number of visits to the website in question (Jarvenpaa et al., 1998). Similarly, Song also noted in an article that past positive experiences have a positive effect on high levels of behavioral belief, which can enhance the individual’s behavioral intention to use technology systems (Song, 2007). In a study concerning the sharing of health information, Bansal et al. suggested that past positive experiences can influence the behavioral intentions of website users to share health information online directly (Bansal and Gefen, 2010). Many studies have shown an influential relationship between trust and attitude (Cook and Wall, 1980; Jones, 1996).

OHCs are consultative communication platforms that facilitate doctor–patient interactions, and these experiences influence users’ levels of trust and dependence on OHCs. When users trust OHCs, they may have a friendlier attitude and exhibit a higher level of willingness to share their personal EHRs. Conversely, previous negative experiences can damage users’ level of trust in OHCs, which in turn may affect their attitudes toward sharing their EHRs. Therefore, past positive experiences may influence the attitudes of patients in OHCs with respect to the action of sharing EHRs.

Simultaneously, patients with past positive experiences in OHCs are bound to be well adapted to OHCs that feature an online environment. In this case, such patients are also more likely to receive more social rewards from the website, doctors and other patients. Therefore, users in OHCs who have had past positive experiences are more likely to receive social rewards. The more past positive experiences that a user has had, the more social rewards they tend to receive.

Perceived behavioral control is itself a hindrance that reflects an individual’s past experiences and expectations, and this factor is necessarily influenced by past positive experiences. Patients with more past experiences of OHCs tend to be more familiar with such websites and to have access to better resources. Furthermore, the resistance such patients encounter when using the website tends to be lower. The more positive a patient’s experiences, the more capable that patient in of behaving in the appropriate manner. In other words, the higher a patient’s level of past positive experience is, the higher that patient’s perceived behavioral control in the context of sharing his or her EHRs.

*H4a*: Past positive experiences have a positive effect on the social rewards received by OHC users.

*H4b*: Past positive experiences have a positive impact on patients’ attitudes toward sharing EHRs in OHCs.

*H4c*: Past positive experiences have a positive effect on patients’ perceived behavioral control when sharing EHRs in OHCs.

#### 1.4.8. Effect of disease severity on attitudes

Disease severity refers to the perceptions of users of OHCs who subjectively experience their health status as good or poor. In addition to personality traits, personal health status also influences one’s attitudes toward one’s personal EHRs. Tisnado et al. investigated the effect of the relationship between demographic characteristics and individual health status on the consistency between real health records and self-shared EHRs (Tisnado et al., 2006). These authors found that this consistency only differed significantly with respect to patient health status. Patients who perceived their health status as poor tend to be more sensitive to their EHR than others. Patients may fear that once their medical health information is disclosed, this information is no longer be confidential (Rindfleisch, 1997). Applying these studies to the online environment, the perceived health status of internet users can influence their attitudes regarding the behavior of sharing their personal EHRs (Bansal and Gefen, 2010).

The primary purpose of users of OHCs is to obtain medical help and facilitate patient communication. Their levels of participation and activity in such communities varies across different states of health. When patients are healthy, they do not need to consult with doctors or contribute content such as their medical experiences. Considering the privacy of EHRs, such patients may reject the possibility of sharing their medical health information. When patients are in poor health, in contrast, they must obtain assistance to improve their health, and a prerequisite for obtaining help is explaining their own condition to the persons from whom they are seeking help; therefore, these patients are forced to share their EHRs autonomously. Simultaneously, the attitudes of such patients toward this behavior tend to change. Hence, we assume that OHC users’ perceptions of disease severity affect their attitudes regarding sharing their personal EHRs.

*H5*: Disease severity has a positive effect on the attitudes of OHC users regarding sharing their EHRs.

#### 1.4.9. Influence of health information sensitivity on attitudes and perceived behavioral control

On the one hand, health information sensitivity refers to the perceived sensitivity of OHC users with respect to their personal EHRs. An individual’s perception of information sensitivity depends on his or her individual characteristics and health status. Previous research concerning privacy has found that consumers’ willingness to disclose personal information is closely related to their levels of information sensitivity (Milne, 1997; Phelps et al., 2000; Malhotra et al., 2004). Wang and Petrison noted that consumer reactions to privacy threats depend on the type of information that marketers are asking them to reveal (Wang and Petrison, 1993). This claim is also supported by the research of Milne, who found that when personal information is collected from people, the eligibility rates of the information they provide vary in accordance with the level of sensitivity involved in the information (Milne, 1997). Concerning online EHRs, it has been argued that the greater the sensitivity of this health information is, the more serious are the privacy concerns that people have when posting it, which in turn affects their attitudes toward such behavior (Simon et al., 2009; Bansal and Gefen, 2010). Thus, even when privacy protection measures are implemented by OHCs, EHR-sensitive patients maintain more negative attitudes regarding EHR sharing than nonsensitive patients.

Perceived behavioral control, on the other hand, refers to the patient’s perceived ability to control or remain in control of EHR sharing behavior. The factors that influence perceived behavioral control during internet use can be summarized in terms of four points: (1) the voluntary nature of user information submission; (2) the privacy settings of the website; (3) the type of information collected by the website; and (4) the transparency of information use by the website. That is, information sensitivity can impact perceived behavioral control. Low sensitivity of the information requested by the website not only reduces the user’s sense of apprehension regarding the possibility of his or her personal information being leaked or shared, thus causing the user to feel a high degree of control regarding such personal information, but also increases users’ levels of perceived control over the act of sharing information and vice versa (Hoadley et al., 2010). Therefore, we hypothesize that when privacy protection measures are implemented by OHCs, patients with greater information sensitivity continue to feel less control with respect to sharing their EHRs.

*H6a*: The sensitivity of health information has a negative effect on the attitudes of OHC users when sharing EHRs.

*H6b*: Health information sensitivity has a negative effect on OHC users’ levels of perceived behavioral control when sharing their EHRs.

#### 1.4.10. Moderating effect of chronic diseases

An increasing number of people tend to obtain health information *via* the internet; meanwhile, many patients have also improved their health conditions by consulting health information on the internet. Chronic diseases are a collective term for a group of diseases that is represented by hypertension, coronary heart disease, diabetes, etc. Such diseases are characterized by long durations, complex causes, high levels of recurrence and difficulty with respect to curing, which prompt patients to seek health information more actively in order to receive help and support. Compared with other disease groups, patients with chronic diseases exhibit higher information needs. Simultaneously, chronic patients’ psychological characteristics are different from those exhibited by patients associated with other disease groups. Some studies have concluded that the psychological characteristics most closely associated with chronic disease patients are anxiety, tension, depression, and negative pessimism (Dyrehag et al., 1998; van Montfort et al., 2017).

Approximately 60% or more of OHC users are chronic disease patients, making OHCs the main way that chronic disease patients are able to obtain and share EHRs. Compared with patients associated with other disease groups, to improve their health and relieve psychological stress, chronic disease patients require more social support and help and exhibit higher demands for information; thus, these patients rely more on and tend to trust OHCs. In addition, the quality and quantity of information is more important in this context than are spiritual satisfaction and material rewards. Therefore, for patients with chronic diseases, social rewards have less influence on their levels of willingness to share EHRs. However, due to their demand for social support and care, chronic patients tend to have a stronger sense of trust in and dependence on OHCs and more positive attitudes regarding EHR sharing behavior; in addition, the role of attitudes and perceived behavioral control on their willingness to share tends to be more pronounced (Andreou et al., 2022).

*H7a*: Chronic diseases have a negative moderating effect on the relationship between social rewards and willingness to share EHRs.

*H7b*: Chronic diseases have a positive moderating effect on the relationship between attitudes and willingness to share EHRs.

*H7c*: Chronic diseases have a positive moderating effect on the relationship between perceived behavioral control and willingness to share EHRs.

#### 1.4.11. Control variables

The control variables included in the model used in this study mainly include the following:

##### 1.4.11.1. Gender

Men and women have different social roles and personality traits; women are usually passive, while men tend to be more active. Moreover, different genders feel and act differently in the same situation (Zhang et al., 2014). Therefore, gender may have an impact on individuals’ willingness to share their EHRs.

##### 1.4.11.2. Age

People of different ages have very different physical conditions and life experiences, so their perceptions and feelings in the same situation may also differ (Reuter et al., 2010). Younger people have a more positive attitudes regarding their experiences, while older people are more conservative. Therefore, age may have an impact on individuals’ willingness to share their EHRs.

##### 1.4.11.3. Level of education

The more educated people are, the better their judgment and cognitive abilities and the more likely they may be to behave differently (Ng and Feldman, 2009). Therefore, educational attainment may affect individuals’ willingness to share their EHRs.

Computer use experience. Computer use experience affects people’s attitudes and decisions regarding objects encountered online. The longer people use computers and the more proficient they are in using computers, the more likely they are to choose to share their information. Therefore, computer use experience may have an impact on individuals’ willingness to share their EHRs.

**Figure 2:**
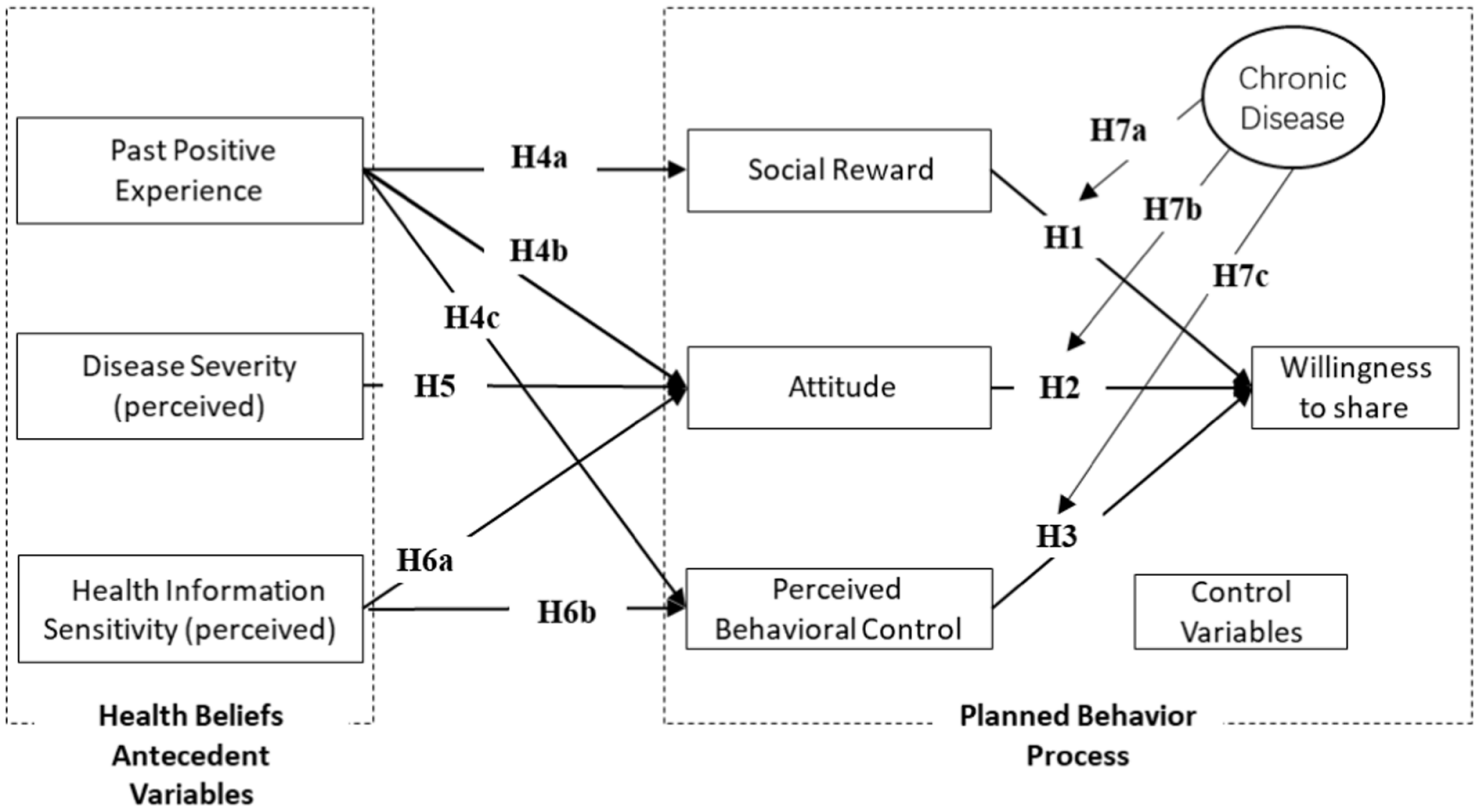
Theoretical framework.

Based on the preceding analysis, this study constructs a model of the factors influencing patients’ levels of willingness to share their EHRs in the context of the privacy protection measures implemented by OHCs. The underlying theoretical framework of the model is the theory of planned behavior, and the antecedent variables are identified based on the health belief model, with chronic disease as the moderating variable. The model structure is shown in Figure 2.

## 2. Materials and methods

### 2.1. Variable measurement and questionnaire design

In this study, the model hypotheses were tested empirically by using a questionnaire research method. First, based on previous research results, this study designed a measurement scale for model construction (see Table 1 for details). The questionnaire included four parts: the first part was used as a basic introduction and to explain the privacy protection measures implemented by OHCs; the second part was intended to allow us to understand respondents’ usage of OHCs; the third part measured the model construction; and the fourth part focused on the basic information of respondents. In the second part of the questionnaire, respondents were asked to indicate their level of agreement or disagreement with the descriptions included on the questionnaire using a 7-point Likert scale. Points 1 to 7 indicated “strongly disagree,” “disagree,” “basically disagree,” “neutral,” “basically agree,” “agree,” and “strongly agree,” respectively. The third part of the indicators allowed us to collected the basic information of respondents, including their genders (GEN), ages (AGE), academic qualifications (EDU), length of internet use (IUT), whether they were suffering from chronic diseases (CD), length of medical website use, and annual frequency of illness.

**Table 1:** Measurement scales.

Constructing	Number	Measurement scales	Sources
Willingness to share	PHID1	I am willing to provide my EHRs to OHC sites and other users (doctors, patients)	Yoo et al. (2013)
	PHID2	I am willing to have my EHRs used by OHC sites and other users (doctors, patients)	
	PHID3	I do not feel bad about disclosing my EHRs to OHC sites and other users (doctors, patients)	
	PHID4	I may share my EHRs with OHC sites and other users (doctors, patients)	
Attitudes	A1	Sharing EHRs with OHC sites and other users (doctors, patients) is a good idea	Kim et al. (2009)
	A2	Sharing EHRs with OHC sites and other users (doctors, patients) is a wise practice	
	A3	I like the practice of sharing EHRs with OHC sites and other users (doctors, patients)	
	A4	The practice of sharing EHRs with OHC sites and other users (doctors, patients) is enjoyable	
Perceptual behavioral control	PBC1	I am able to control the EHRs that I post and share in the OHC	Kim and Kankanhalli (2009)
	PBC2	I have the necessary resources to reduce the likelihood of adverse outcomes from posting and sharing EHRs in OHCs	
	PBC3	I have the necessary knowledge to address the problems that can arise from posting and sharing EHRs in OHCs	
Social rewards	SR1	I feel that using an OHC will satisfy my social needs (e.g., for money, respect, and social status) to some extent	Jiang et al. (2013)
	SR2	I believe that participating in interactions *via* the OHC improves my interpersonal relationships with the site and its users (doctors, patients)	
	SR3	I believe that I can derive spiritual pleasure and satisfaction from participating in an interactive OHC	
Past positive experiences	PPE1	In the past, OHCs and interactions with their users (doctors, patients) have been useful to me	Song (2007)
	PPE2	In the past, I have benefited from OHCs and their users (doctors, patients) a great deal	
	PPE3	In the past, I have frequently had positive interactions with OHCs and their users (doctors, patients)	
Disease severity	DS1	My body rarely suffers from prolonged illness or discomfort	Bansal and Gefen (2010)
	DS2	I do not believe that I have any chronic illnesses in my body	
	DS3	I believe that my general health is very good	
Health information sensitivity	IS1	I feel that the level of sensitivity of the EHRs requested from me by the OHC is not sensitive/sensitive	Bansal and Gefen (2010)
	IS2	I believe that the level of sensitivity of EHRs shared by other users in OHCs is not sensitive/sensitive	
	IS3	I feel that the level of sensitivity of the EHRs that I share in OHCs is not sensitive/sensitive	

### 2.2. Sample data collection

#### 2.2.1. Prestudy and questionnaire validity test

The validity of the questionnaire must be tested prior to the formal mass distribution of the questionnaire. In this study, the questionnaire was modified and improved in accordance with the results of this test in terms of both content validity and structural validity.

First, the content validity of the questionnaire was examined. The questionnaire was evaluated by other scholars to highlight its shortcomings. Subsequently, the questionnaire was simplified and modified to address these shortcomings. The content of the questions concerning perceived behavioral control and social rewards was largely simplified. The wording of some questions was also modified, such as by replacing “a lot” with “very much.”

Subsequently, the questionnaire was tested to examine its construct validity. During this stage, a small-scale presurvey was conducted mainly *via* the internet, and online questionnaires were distributed to OHC users. Eighty questionnaires were distributed, and 59 valid questionnaires were returned.

##### 2.2.1.1. Convergent validity test

The indicators used to test convergent validity were factor loading, Cronbach’s alpha, composite reliability, and average variance extraction (AVE). The relevant literature has indicated that the following conditions, shown in Table 2, should be met during the convergent validity test.

**Table 2:** Aggregate validity test table.

Indicators	Conditions to meet	Literature sources
Factor loading coefficient	>0.75	Chin et al. (2003) and Mun and Davis (2003)
Cronbach’s α	>0.70	
Overall confidence coefficient	>0.70	
Mean variance extraction	>0.50	

Factor analysis was first conducted with respect to each construct by using software Smart PLS, and factor loadings less than 0.75 were removed. Subsequently, the retained prestudy data were reanalyzed for convergent validity (Appendix A), and it was found that the Cronbach’s alpha coefficient and the composite reliability coefficient of each construct were greater than 0.75 and that the mean variance extracted was greater than 0.6. Therefore, each construct exhibited good convergent validity with respect to its measure (Chin et al., 2003).

##### 2.2.1.2. Discriminant validity test

Discriminant validity tests are usually measured in terms of two indicators: (1) the correlation coefficients among constructs are less than the arithmetic square roots of the mean variance extracted from the constructs themselves and (2) the cross loadings for each measure on a construct are greater those on the other constructs. Discriminant validity tests were conducted on the prestudy data using Smart PLS (Appendix B). The results showed that the correlation coefficients for each construct were smaller than the diagonal data, i.e., the arithmetic square root of the mean variance extracted for the constructs themselves. In addition, the factor loadings of each measure on the constructs were greater than those on other constructs. Therefore, there was good discriminant validity among the constructs.

In summary, this prestudy questionnaire exhibited good convergent validity and discriminant validity, and the measures of each construct were valid for large-scale distribution to collect sample data for empirical research.

#### 2.2.2. Questionnaire distribution and collection

The results of the prestudy and questionnaire validity tests indicated that this research questionnaire was valid; some of the questions with small factor loadings were deleted, and the remaining questions were renumbered. Subsequently, the final research questionnaire was designed (see Appendix C for details). A large-scale distribution of the final questionnaire was conducted for the purposes of this study. The target population mainly included patient users of OHCs, and there were no restrictions concerning particular communities and websites. The questionnaires were mainly created *via* the Questionnaire Star platform and were sent to respondents in the form of an internet link; a few questionnaires were distributed on paper. A total of 331 questionnaires were distributed and 234 valid questionnaires were collected; accordingly, the effective return rate of the questionnaire was 70.7%.

#### 2.2.3. Descriptive statistical analysis of the sample

A total of 234 valid questionnaires were collected for this study, and the collected samples were statistically described in terms of seven aspects, including gender, age, education, length of internet use, length of online medical website use, frequency of illness per year, and whether the respondent suffered from a chronic disease, as shown in Table 3.

**Table 3:** Descriptive statistics of the sample.

Statistical indicator	Statistical variable	Number of respondents	Frequency
Gender	Male	85	36.3%
	Female	149	63.7%
Age	≤20 years old	3	1.28%
	21–30 years old	101	43.16%
	31–40 years old	112	47.86%
	41–50 years old	16	6.84%
	51–60 years old	1	0.43%
	Over 60 years old	1	0.43%
Academic qualifications	Junior high school or below	0	0.00%
	High school	8	3.42%
	Specialized training school	21	6.34%
	Bachelor’s degree	166	70.94%
	Master’s degree	34	14.53%
	PhD or above	5	2.14%
Length of internet use	≤1 year	0	0.00%
	2–4 years	18	7.69%
	5–7 years	63	26.92%
	8–10 years	75	32.05%
	More than 10 years	78	33.33%
Length of online medical website use	≤1 year	52	22.22%
	2–3 years	129	55.13%
	4–5 years	43	18.38%
	6–7 years	8	3.42%
	More than 7 years	2	0.85%
Annual frequency of illness	≤1 time	38	16.24%
	2–3 times	144	61.54%
	4–6 times	44	18.80%
	7–10 times	6	2.56%
	More than 10 times	2	0.85%
Suffering from a chronic disease	YES	87	37.18%
	NO	147	62.82%

According to data drawn from the “China Online Medical Market Research Report” by Enfodesk, the ratio of male to female users of online medical websites in China is 49.7%:50.3%, with a higher proportion of female users; youths aged 19–35 are the main group of such users, accounting for 69.1% of the total population; the majority of users have an undergraduate level of education or higher, accounting for 73.9% of the total; and patients with chronic diseases account for a large proportion of the population. By comparing the sample statistics of this study with the report from Enfodesk, it can be seen that the proportions basically match. This fact indicates that the sample is representative of the overall population to a certain degree. Moreover, approximately 80% of users included in the sample have used online medical websites for more than 2 years, making the sample highly representative.

#### 2.2.4. Test for normal distribution of sample data

Bentler et al. noted that a prerequisite of structural equation modeling analysis is that the sample dataset must satisfy the requirements of normal distribution (Bentler and Chou, 1987). In this study, the absolute values of skewness and kurtosis should be less than the threshold of 2 and 5, respectively, to determine whether the sample data satisfied the requirements of normal distribution according to the criteria proposed by Bentler. Descriptive statistical analysis was performed on the sample data using SPSS 19.0 software (Appendix D). The results showed that the absolute values of skewness of the sample data were all less than 2 and that the absolute values of kurtosis were all less than 5. Therefore, the sample data satisfied the normal distribution requirement and were suitable for structural equation analysis.

The structural equation analysis conducted for this study consisted of two steps: measurement model analysis and structural model analysis. Measurement model analysis was used to test the reliability of the sample data, while the structural model was used to test the model hypothesis.

## 3. Results

### 3.1. Measurement model analysis

A measurement model analysis was first conducted to examine the convergent validity and discriminant validity of the sample data. The results of the convergent validity analysis (Table E.1 in Appendix E) showed that the factor loadings of each measure were greater than 0.75, the Cronbach’s α coefficients for each construct were greater than 0.8, and the composite reliability coefficients and mean variance extracted were greater than 0.7, thus indicating that the convergent validity of the measurement model was good. The discriminant validity test results showed that the arithmetic square root of the mean variance of each construct was greater than the correlation coefficient (Table E.1 in Appendix E) and that the factor loading coefficients of each construct on its measurement items were greater than the loadings on those of others (Table E.2 in Appendix E). Therefore, the discriminant validity of the measurement models in this study was acceptable.

### 3.2. Structural model analysis

The measurement model analysis described above demonstrated that the sample data of this study exhibited good reliability and validity. Subsequently, structural model analysis was conducted to validate the model hypotheses. This analysis consisted of two main steps. First, the validation of the base model was used to test the significance of the relationships among the constructs. Second, the validation of the complete model after adding the moderating variables was used to observe the moderating effect of chronic diseases.

**Figure 3:**
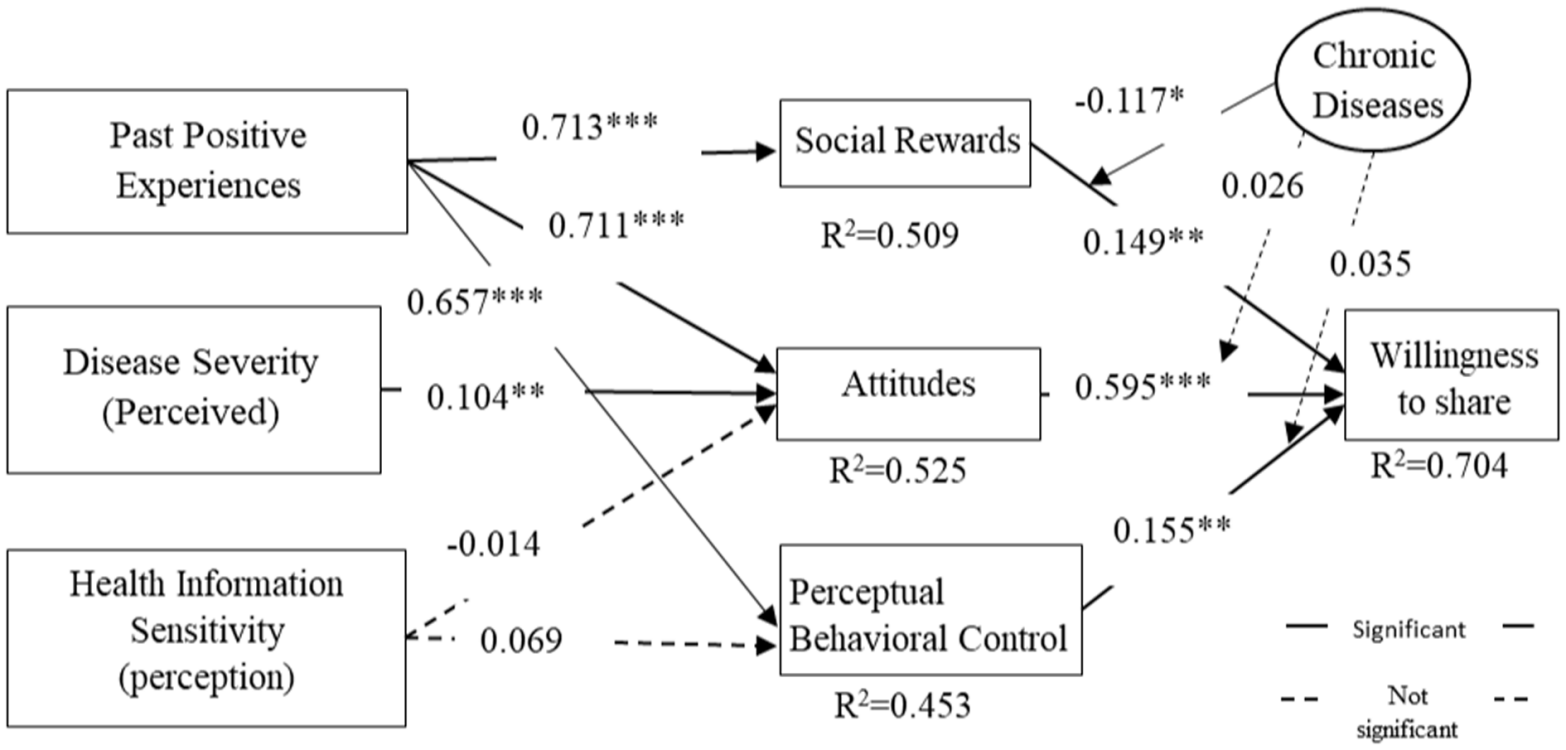
Schematic diagram of the complete model analysis results. *p < 0.1, **p < 0.05, ***p < 0.01.

The base model test was first conducted by ignoring the moderating variables and considering only the effect of the antecedent variable on the independent variable and the effect of the independent variable on the dependent variable; this process was mainly used to verify Hypotheses H1–H6. The results of the base model validation are shown in Appendix F. The complete model validation was conducted on the basis of the base model, and the moderating effect of chronic disease was then added to the base model to test the significance of the moderating effect of chronic disease, as shown in Table 4 and Figure 3.

**Table 4:** Complete model validation results.^a^

Hypothesis	Route	Coefficient	*T*-test value	Significance	Degree of variance explained R2
H1	Attitude → willingness to share	0.595	7.633	***	0.704
H2	Perceptual behavioral control → willingness to share	0.155	1.758	**	
H3	Social rewards → willingness to share	0.149	1.754	**	
H7a	Social rewards * chronic diseases → willingness to share	−0.117	1.434	*	
H7b	Attitudes * chronic diseases → willingness to share	0.026	0.351	NS	
H7c	Perceptual behavioral control * chronic diseases → willingness to share	0.035	0.414	NS	
Control variables	Gender → willingness to share	−0.046	1.178	NS	
	Age → willingness to share	0.03	0.718	NS	
	Level of education→ willingness to share	−0.013	0.312	NS	
	Computer use experience → willingness to share	0.02	0.502	NS	
H4a	Past positive experiences → social rewards	0.713	22.512	***	0.509
H4b	Past positive experiences → attitudes	0.711	16.568	***	0.525
H5	Disease severity → attitudes	0.104	1.834	**	
H6a	Health information sensitivity → attitudes	−0.014	0.195	NS	
H4c	Past positive experiences →perceptual behavioral control	0.657	15.587	***	0.453
H6b	Health information sensitivity → perceptual behavioral control	0.069	1.152	NS	

a *** Indicates significance at the 0.01 confidence level; ** indicates significance at the 0.05 confidence level; * indicates significance at the 0.1 confidence level; NS indicates non significance.

### 3.3. Hypothesis testing results

The results of the structural equation analysis produced the hypothesis testing results of this study, as shown in Table 5.

**Table 5:** Hypothesis validation results.

Number	Hypothetical propositions	Result
H1	Social rewards have a positive effect on the intention to share their EHRs	Supported
H2	Attitudes have a positive effect on the intention to share their EHRs	Supported
H3	Perceived behavioral control has a positive effect on the intention to share their EHRs	Supported
H4a	Past positive experiences have a positive effect on OHCs users’ social rewards	Supported
H4b	Past positive experiences have a positive impact on patients’ attitude toward sharing their EHRs in OHCs	Supported
H4c	Past positive experiences have a positive effect on patients’ perceived behavioral control with respect to sharing their EHRs in OHCs	Supported
H5	Disease severity has a positive effect on the attitudes of OHCs users toward sharing their EHRs	Supported
H6a	The sensitivity of health information has a negative effect on the attitudes of OHC users when sharing their EHRs	Unsupported
H6b	Health information sensitivity has a negative effect on OHCs users’ perceived behavioral control with respect to sharing their EHRs	Unsupported
H7a	Chronic diseases have a negative moderating effect on the relationship between social rewards and willingness to share EHRs	Supported
H7b	Chronic diseases have a positive moderating effect on the relationship between attitudes and willingness to share EHRs	Unsupported
H7c	Chronic diseases have a positive moderating effect on the relationship between perceived behavioral control and willingness to share EHRs	Unsupported

## 4. Discussion

### 4.1. Discussion of model estimation results

This study investigated the factors influencing patients’ willingness to share their EHRs after OHCs implement privacy protection measures based on the TPB model alongside the antecedents defined by the health belief model, constructed a theoretical model of the factors influencing willingness to share, and proposed nine significance hypotheses and three moderating hypotheses. Analysis of the empirical results showed that the significance hypotheses were all valid with the exception of H6a and H6b, while only H7a was valid among the moderating effect hypotheses.

According to the base model analysis, 52.5% of the variance in attitudes was explained, 50.9% of the variance in social reward was explained, 45.3% of the variance in perceived behavioral control was explained, and 69.4% of the variance in willingness to share was explained by attitudes, perceived behavioral control, and social rewards, thus indicating that the model has good explanatory power and that the independent variables included in the model are the main factors influencing the dependent variable. According to the complete model analysis, the inclusion of the moderating effects of chronic diseases increased the degree of explanation of willingness to share by 1.0%, thus validating H7a.

#### 4.1.1. Analysis of the effects

According to the results of this study, willingness to share is determined by three factors, namely, attitude, perceived behavioral control and social rewards, and the effect of all these factors are all significantly positive. In other words, after patients are informed of the privacy protection measures implemented by OHCs, their positive attitudes toward the OHCs, their higher levels of perceived control and the greater social rewards they receive all cause an in their willingness to share; this result is consistent with planning theory. Moreover, past positive experience indirectly affect patients’ willingness to share *via* its positive effects on social rewards, attitudes and perceived behaviors. However, health information sensitivity has no significant effect on attitude and perceived behavioral control and thus has no effect on willingness to share.

Past positive experience represents patients’ past positive experiences with participation in OHCs, and these experiences influence patients’ current perceptions of such websites and their sharing behavior. When patients have had past experiences with OHCs that were positive or pleasant, they may exhibit more precise and optimistic perceptions of the mental and psychological rewards that can be obtained *via* OHCs when deciding whether to share their health information online, and their attitudes regarding the ways in which they may share their health information are also favorable or approving. Meanwhile, patients with high levels of perceived behavioral control, namely, patients who perceive that they have mastered the skills or abilities that are necessary to perform certain behaviors, tend to be more willing to share their health information. In contrast, when patients perceive low rewards from sharing and are suspicious that their ability is insufficient to cope with the consequences of sharing, their willingness to share in OHCs is limited due to their negative past experiences. Moreover, it should be noted that past positive experiences have a high level of impact on social rewards, attitudes and perceived behavioral control. In other words, past positive experience is the most significant of the many factors that influence willingness to share, which means that patients rely strongly on past experience when considering participation in OHCs, and patients who have had positive experience are more likely to use OHCs for EHR sharing and communication when offered privacy protection measures by OHCs.

Disease severity refers to patients’ perceptions of their individual health situations. The ultimate goal of patients’ usage of OHCs is to improve their individual health, and patients have an intuitive sense of their own diseases, which can influence patients’ attitudes toward health information sharing behavior. When patients consider themselves to be seriously ill, they tend to be more positive with respect to sharing their health information to obtain support from doctors or other patients. Conversely, when a patient’s disease is moderate, his or her attitude toward such sharing tends to be less positive. This difference indicates that disease severity has a positive effect on attitudes and an indirect influence on willingness to share, but the effect of disease severity is weaker than the effect of past positive experience.

Health information sensitivity refers to the patients’ perceptions of the degree to which their health information is sensitive, which pertains to their concern for personal privacy. Our research hypothesizes that information sensitivity has a negative impact on attitudes and perceived behavioral control. However, the results of our empirical research show that these two hypotheses are invalid. These findings indicate that health information sensitivity has no indirect effect on willingness to share. This lack of an effect might be due to the fact that the influential role played by health information sensitivity is weakened by privacy protection measures, indicating that health information sensitivity has no effect on attitudes and perceived behavioral control and thus no influence on willingness to share. However, this study did not verify this hypothesis.

Social reward is a new variable introduced in this study as a modification of the TPB model; it refers to the rewards such as money, materials, or the improvement of interpersonal relationships that can be obtained *via* participation in a website, and it is used as a substitute for the subjective normative variable “social pressure.” Social rewards, alongside attitudes and perceived behavioral control, jointly influence willingness to share. This impact indicates that social rewards such as material, monetary rewards or friendly interpersonal relationships online are also one of the purposes for which patients participate in OHCs in addition to the improvement of their personal health situations; both a sense of pleasant engagement and positive experiences can promote sharing behavior.

Attitude and perceived behavioral control are both included in the original construction of the TPB model, and these factors have positive impacts on willingness to share, which is consistent with the findings of previous studies; in addition, OHCs can improve attitudes and perceived behavioral control by shaping past positive experiences and disease severity to enhance patients’ willingness to share.

According to the preceding analysis, this study explored the explanatory power of the TPB model in the context of the privacy protection measures implemented by OHCs. In this context, the TPB model was revised by introducing the variable of social rewards to ensure better explanatory power. Moreover, the influence of antecedent variables such as past positive experience and disease severity on willingness to share were recognized.

#### 4.1.2. Analysis of the moderating effect

Chronic diseases are characterized by long disease courses, complex causes, high levels of recurrence and difficulty with respect to curing, and patients with chronic diseases thus exhibit unique psychological characteristics and behavioral patterns. This study includes chronic disease as a moderating variable in the theoretical model to determine whether chronic diseases influence health information sharing behavior in the context of OHCs, and the results show that chronic diseases have only a negative moderating effect on the relationship between social rewards and willingness to share.

**Figure 4:**
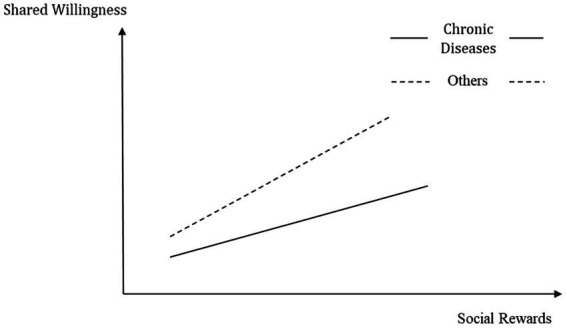
Schematic diagram of the regulatory effect of chronic diseases.

As shown in Figure 4, for patients with chronic diseases, the effect of social rewards on willingness to share is decreased. This decrease also reduces patients’ willingness to share to a certain degree. This effect is due to the fact that patients with chronic diseases have greater information needs and are more motivated to participate in OHCs; compared to their information needs and the urgency of improving their health status, social rewards are less important to them. Accordingly, when such patients share health information, social rewards do not significantly influence their willingness to share, and so the increase in behavioral intentions typically associated with social rewards is more moderate in such a case.

### 4.2. Theoretical implication

Based on the TPB model, this paper analyses the factors influencing patients’ willingness to share their EHRs in the context of the privacy protection measures implemented by OHCs from the perspectives of patients and the health belief model. The purpose of this study is to enrich and improve the extant theoretical research concerning information sharing.

First, this research is grounded in the study of the strongly privacy-related behaviors associated with EHR sharing in OHCs and uses an innovative method to explore decisions pertaining to EHR sharing behavior in the context of the privacy protection measures implemented by OHCs. Instead of the widely used privacy calculus theory, this study utilizes the theory of planned behavior to analyze the behavioral patterns exhibited patients who use OHCs and engage in EHR sharing in further detail, thus avoiding the concern of a high privacy factor while simultaneously expanding the range of influencing factors. Based on TPB, this research investigates the roles played by attitudes, perceived behavioral control, and social rewards in patients’ willingness to share EHRs. In particular, the substitution of the original variable of subjective norms in TPB by the introduction of the variable “social rewards” contributes to the development of the theory of planned behavior with respect to application to the field of online health care. The research results verify that attitudes and perceived behavioral control have positive effects on personal behavioral intention, which is consistent with the theory of planned behavior. Furthermore, this research reveals the fact that social rewards also influence patients’ willingness to share their EHRs, which highlights the influence of social pressure, thus indicating that patients consider the material rewards, online interpersonal relationships, mental pleasure and satisfaction and respect or gratitude from others. The more valuable these social rewards are, the more willing patients are to share their EHRs. This finding illustrates that although patients continue to value the ability to control their privacy (i.e., they take into account their possible losses), the perceived value of shared rewards increases after OHCs implement privacy protection measures.

Second, this research identifies health belief patterns in the context of actors’ (i.e., patients’) personal perceptions alongside the characteristics of OHCs and constructs antecedent variables for the TPB model to improve the explanatory power of the model, thus providing new ideas and methods that can be used by similar studies in the future. This research ultimately introduces disease severity, information sensitivity, and past positive experience to the model as antecedent variables based on these health belief patterns and explores the mechanisms by which they operate on patients’ health information sharing behavior empirically. The results show that past positive experience significantly and positively influences attitudes, perceived behavioral control and social rewards and that disease severity significantly and positively influences attitudes, which jointly and indirectly influence patients’ willingness to share EHRs. However, information sensitivity has no impact on willingness to share, thus indicating that the privacy protection measures implemented by OHCs can diminish the information sensitivity of privacy-sensitive patients.

Furthermore, this study also investigates the moderating effect of chronic diseases on patients’ willingness to share their EHRs. Populations with different diseases have different levels of sensitivity to the social rewards that are generated by OHCs. Compared to other disease populations, chronic disease patients have lower levels of sensitivity to social rewards, thus reflecting the uniqueness of their psychological characteristics and behavioral patterns to a certain degree.

### 4.3. Practical implications

In practical terms, this study explores the factors influencing patients’ willingness to share their EHRs and provides an in-depth analysis of the mechanisms operative in their actions, which can assist OHC providers in improving their understanding of the values and behavioral patterns of patient users and in distinguishing between chronic disease patients and other disease patients. Subsequently, we can utilize the findings reported above to (1) optimize the service and experiences associated with OHCs, thus promoting the sustainable development of OHCs, (2) provide appropriate incentives to promote sharing behavior and enrich medical resources and information, (3) alleviate the asymmetry of information and resources and assist other patients in obtaining proper treatment swiftly, and (4) alleviate the problem of shortages and uneven distributions of health resources, help patients realize online consultation and treatment, and provide comments and suggestions for the development of OHCs and medical and health care services in China.

When patients share their EHRs in OHCs, they have an intuitive perception of the severity of their own diseases and a strong degree of reliance on previous experiences. Past experiences determine patients’ perceptions of the social rewards that can be obtained *via* OHCs and their own ability to control their EHR sharing behaviors, and such experiences also influence patients’ attitudes toward the behaviors associated with sharing health information. Moreover, information sensitivity has no impact on patients’ sharing behaviors. This outcome reflects the importance of the privacy protection measures implemented by OHCs and the necessity of informing patients of those measures, which can both significantly impact patients’ EHR sharing behavior and facilitate OHC service providers in the tasks of developing programs and measures to incentivize patient contribution behaviors and promoting OHC development.

In addition, OHC service providers must offer physical or mental rewards to users who exhibit a high rate of contributions to ensure a positive use experience. It is also advisable to implement certain evaluation functions to allow other users to comment on or like patient contributions. Certain incentive measures can be adapted to encourage friendly relationships between patients and doctors and between patients and other patients, thus allowing patients to experience care and respect from others. Meanwhile, the moderating effect of chronic diseases suggests that OHC providers should treat the group of chronic disease patients differently from other patient populations to improve the survival rates and service experiences of chronic disease patients. OHC service providers may not be as effective in increasing EHR sharing behaviors by offering lucrative rewards to patients with chronic diseases; measures other than social rewards, such as charity clinics, patient meetings and offline events, may be more effective in this context.

### 4.4. Limitations

The study reported in this paper has certain limitations and shortcomings:

1. The sample used in this study is relatively small and unevenly distributed. Users of OHCs are a large group and are distributed across various cities throughout the country. Although the data used in this study were obtained *via* the internet, the sample size was insignificant compared to the large group of OHC users, and the sample did not cover all cities, which may affect the results of this study.
2. The factors influencing patients’ willingness to share their EHRs are complicated and multifaceted. This study summarizes only three influencing factors, which represents a limitation of this study and prevents us from providing a comprehensive explanation of patients’ EHR sharing behavior.
3. When verifying the moderating effect of chronic diseases, the sample was differentiated only in terms of an indicator of the presence of a chronic disease, which was a quite simple measure. Future studies can consider different types of disease and employ richer measures to verify the moderating effect of disease types on patients’ behavioral patterns, such as by reference to mental health (Lu et al., 2021) or albinism (Bi et al., 2020).

## 5. Conclusion

This study investigates patients’ willingness to share EHRs in OHCs and focuses on the context of the realistic developmental stages at which OHCs implement privacy protection measures. This study combines the health belief model and the theory of planned behavior to construct a model of the factors that influence patients’ willingness to share EHRs in OHCs, and the TPB model is modified and extended to improve the explanatory power of the proposed model. The variables of disease severity, health information sensitivity, and past positive experience are introduced as antecedent variables of the model from the perspective of patients’ perceptions of three relevant aspects, i.e., their health status, health information sensitivity, and community website participation experiences, as well as the main factors influencing patients’ willingness to share EHR highlighted by this study. In addition, chronic diseases were added as regulating variables in the model to investigate the regulating effect of disease type in this context. The relevant findings also provide theoretical and practical suggestions for promoting patients’ full participation in OHCs and encouraging OHC development from a privacy perspective.

## Data availability statement

The original contributions presented in the study are included in the article/Supplementary material, further inquiries can be directed to the corresponding author.

## Author contributions

SG contributed to the research idea and paper writing. YD contributed to the paper writing and revised version. YL contributed to the data analysis and paper writing. BS contributed to the data analysis. All authors contributed to the article and approved the submitted version.

## Funding

This research was funded by the National Natural Science Foundation of China (72101090, 72171152, 71925002, 71801062, 71731006, and 71801096), the Special Fund Project for Scientific and Technological Innovation (Soft Science) of Guangdong Province (2022A1515011620, and 2022A1515011983), Guangdong Philosophy and Social Sciences (GD21YGL09), and the China Postdoc Science Foundation (2021M701242).

## Conflict of interest

The authors declare that the research was conducted in the absence of any commercial or financial relationships that could be construed as a potential conflict of interest.

## Publisher’s note

All claims expressed in this article are solely those of the authors and do not necessarily represent those of their affiliated organizations, or those of the publisher, the editors and the reviewers. Any product that may be evaluated in this article, or claim that may be made by its manufacturer, is not guaranteed or endorsed by the publisher.

## Supplementary material

The Supplementary material for this article can be found online at: https://www.frontiersin.org/articles/10.3389/fpsyg.2022.1047980/full#supplementary-material
