# Supplementary material for: Sharing intention of electronic health records in online health communities: Patients’ behavioral decisions in the context of privacy protection measures
Source: Front Psychol. 2022 Dec 22;13:1047980. doi: 10.3389/fpsyg.2022.1047980 (PMC9813434; doi:10.3389/fpsyg.2022.1047980)
Supplement: Supplementary file 1 [file Data_Sheet_1.pdf]

Appendix A

Table A Pilot analysis Convergent Validity Test

Construction	Measurement indicators	Cronbach's α Coefficient	Overall Confidence Coefficient	Mean Variance Extraction
Willingness to share	PHID2, PHID3, PHID4 (Removing PHID1 with small factor loadings)	0.863	0.916	0.785
Attitude	A1, A2, A3, A4	0.940	0.957	0.848
Perceptual Behavioral Control	PBC1, PBC2, PBC3	0.890	0.932	0.821
Social Rewards	SR1, SR2, SR3	0.810	0.887	0.724
Past Positive Experiences	PPE1, PPE2, PPE3	0.820	0.893	0.735
Severity of disease	DS2, DS3 (Removing DS1 with smaller factor loadings)	0.910	0.922	0.845
Information Sensitivity	IS1, IS2, IS3	0.884	0.917	0788

Appendix B

Table B Table of Correlation Coefficients for the Pre-Survey

Construction	Attitude	Severity of disease	Information Sensitivity	Perceptual Behavioral Control	Willingness to share	Past Positive Experiences	Social Rewards
Attitude	**0.921**						
Disease Severity	-0.095	**0.925**					
Information Sensitivity	0.239	-0.014	**0.888**				
Perceptual Behavioral Control	0.69	-0.078	0.092	**0.906**			
Willingness to share	0.793	-0.083	0.199	0.597	**0.886**		
Past Positive	0.6	-0.185	0.144	0.514	0.651	**0.857**	

Experiences	91						
Social	0.6	-0.147	0.168	0.564	0.636	0.679	**0.851**
Rewards	83						

Note: The diagonal data are the arithmetic square root of the corresponding constructed AVE

Appendix C

Welcome to this survey on the willingness of OHC users to share EHRs. Thank you very much for taking the time to fill out the survey. This survey is for academic research purposes to examine your willingness to share EHRs, such as your medical history, medications taken, medical procedures and experiences, when using the internet or online medical websites. We promise that the answers you provide will be used for academic research purposes only and that all information you provide will be kept completely confidential. Please answer according to your real experience and feelings and select the option after the question to indicate your agreement/disagreement with our description.

Among them, EHR mainly involves one's physical condition, personal medical history, medications taken, medical treatment process and experience.;; OHCs mainly includes BBS, forums and other interactive communities set up by medical and health websites such as Good Doctor Online, SeekMedicine.com, Family Doctor Online, 39Health.com, etc.

Prerequisite introduction

The existing OHCs have already implemented corresponding privacy protection for users' EHRs, including hiding patients' personal information (such as name/gender/address/contact information), only doctors can see the examination results such as images, intelligent identification of offline treatment information (hospital/department/doctor, etc.), etc. Please answer with known protection

I. Have you ever used an OHC (not limited to a specific community, any one will do)?

□ Yes □ No

II. Your experience and feelings.

	My willingness to disclose my EHR to the OHC can be described as	**Strongly Disagree**	**Disagree**	**Somewhat disagree**	**Neutral**	**Somewhat Agree**	**Agree**	**Very Agree**
**PHID1**	I am willing to have my EHR used by OHC sites and other users (doctors, patients)	1	2	3	4	5	6	7

**PHID2**	I do not feel bad about disclosing my EHR to OHC sites and other users (doctors, patients)	1	2	3	4	5	6	7
**PHID3**	I am willing to have my EHRs used by OHC sites and other users (doctors, patients)	1	2	3	4	5	6	7
	My attitude toward disclosing my EHR to OHCs is.	**Strongly Disagree**	**Disagree**	**Somewhat disagree**	**Neutral**	**Somewhat Agree**	**Agree**	**Very Agree**
**A1**	Sharing EHR to OHC sites and other users (doctors, patients) is a good idea	1	2	3	4	5	6	7
**A2**	Sharing EHR with OHC sites and other users (doctors, patients) is a wise practice	1	2	3	4	5	6	7
**A3**	I like the practice of sharing EHR to OHC sites and other users (doctors, patients)	1	2	3	4	5	6	7
**A4**	The practice of sharing EHR to OHC sites and other users (doctors, patients) is enjoyable	1	2	3	4	5	6	7
	With regard to the possible adverse consequences of disclosing EHR, I believe that	**Strongly Disagree**	**Disagree**	**Somewhat disagree**	**Neutral**	**Somewhat Agree**	**Agree**	**Very Agree**
**PBC1**	I am able to control the EHR I	1	2	3	4	5	6	7

	post and share in the OHC							
**PBC2**	I have the necessary resources to reduce the likelihood of adverse outcomes from posting and sharing EHR in OHCs	1	2	3	4	5	6	7
**PBC3**	I have the necessary knowledge to address the problems that can arise from posting and sharing EHR in OHCs	1	2	3	4	5	6	7
	The social rewards that I can get from using the OHC are	**Strongly Disagree**	**Disagree**	**Somewhat disagree**	**Neutral**	**Somewhat Agree**	**Agree**	**Very Agree**
**SR1**	I feel that using an OHC will to some extent satisfy my social needs (e.g., money, respect, social status)	1	2	3	4	5	6	7
**SR2**	I believe that participating in interactions with the OHC promotes my interpersonal relationships with the site and its users (doctors, patients)	1	2	3	4	5	6	7
**SR3**	I believe I can derive spiritual pleasure and satisfaction from	1	2	3	4	5	6	7

	participating in the interactive OHC							
	My past experiences with OHCs can be described as	**Strongly Disagree**	**Disagree**	**Somewhat disagree**	**Neutral**	**Somewhat Agree**	**Agree**	**Very Agree**
**PPE1**	In the past, OHCs and interaction with their users (doctors, patients) have been useful to me	1	2	3	4	5	6	7
**PPE2**	In the past, I have benefited from OHCs and their users (doctors, patients) a great deal	1	2	3	4	5	6	7
**PPE3**	In the past, I have often had positive interactions with OHCs and their users (doctors, patients)	1	2	3	4	5	6	7
	I believe that my physical health is：	**Strongly Disagree**	**Disagree**	**Somewhat disagree**	**Neutral**	**Somewhat Agree**	**Agree**	**Very Agree**
**DS1**	I don't think my body has any medical conditions	1	2	3	4	5	6	7
**DS2**	I think my general health is very good	1	2	3	4	5	6	7
	Description of health information sensitivity：	**Very Insensitive**	**Insensitive**	**Somewhat Insensitive**	**Neutral**	**Somewhat Sensitive**	**Sensitive**	**Very Sensitive**
**IS1**	I am concerned that the medical information submitted online may be misused	1	2	3	4	5	6	7

**IS2**	I'm worried about other people being able to see my medical information online	1	2	3	4	5	6	7
**IS3**	I worry that the health information I submit online could be used by others in an unexpected way	1	2	3	4	5	6	7

III. Your basic information.

Gender (**GEN**):	□ Male □ Female
Age (**AGE**):	□ ≤20 □21-30 □ 31-40 □ 41-50 □ 51-60 □ Greater than 60
Education (**EDU**):	□ Junior high school and below □ High School □ Specialized Training School □ Bachelor's degree □ Master's degree □ PhD and above
Hours of internet use (**UIT**):	□≤1 year □2-4 years □5-7 years □8-10 years □More than 10 years
Length of time spent on online medical websites:	□≤1 year □2-3 years □4-5 years □6-7 years □ More than 7 years
How often you get sick each year:	□ Less than 1 time □2-3 times □4-6 times □7-10 times □ More than 10 times
Do you suffer from any of the following diseases (CD, multiple choice)?	□ Hypertension □ Diabetes □ Coronary artery disease □ Myocardial infarction □ Other chronic diseases □ None

Appendix D

Table D Sample Normal Distribution Test

Measurement Indicators	Minimum Value	Maximum value	Mean value	Standard deviation	Skewness	Kurtosis
**PHID1**	1	7	5.06	1.454	-0.759	0.047
**PHID2**	1	7	4.85	1.549	-0.682	-0.334
**PHID3**	1	7	5.03	1.490	-0.932	0.249
**A1**	1	7	4.68	1.404	-0.695	0.007
**A2**	1	7	4.70	1.541	-0.464	-0.389
**A3**	1	7	4.71	1.527	-0.461	-0.314
**A4**	1	7	4.54	1.600	-0.506	-0.465
**PBC1**	1	7	5.03	1.386	-0.844	0.038
**PBC2**	1	7	4.67	1.529	-0.552	-0.603
**PBC3**	1	7	4.74	1.515	-0.591	-0.469
**SR1**	1	7	4.60	1.430	-0.465	-0.475
**SR2**	2	7	5.23	1.140	-0.770	0.537
**SR3**	1	7	5.10	1.343	-0.821	0.552
**PPE1**	2	7	5.58	1.165	-0.905	0.842
**PPE2**	1	7	5.21	1.277	-0.866	0.679
**PPE3**	1	7	5.33	1.346	-0.845	0.500
**DS1**	1	7	5.10	1.439	-0.831	0.190
**DS2**	1	7	5.13	1.444	-0.941	0.072
**IS1**	1	7	4.75	1.705	-0.454	-1.000
**IS2**	1	7	4.61	1.791	-0.465	-1.013
**IS3**	1	7	4.84	1.690	-0.544	-0.764
**GEN**	1	2	1.66	0.476	-0.664	-1.576
**AGE**	1	4	2.61	0.603	0.300	-0.551
**EDU**	3	7	5.06	0.639	0.066	2.857
**UIT**	2	5	3.90	0.945	-0.397	-0.831
**CD**	0	1	0.38	0.487	0.501	-1.768

Appendix E

Table E.1 Convergent Validity Test

Construction	Measurement Indicators	Standardized Factor Loading Coefficients	Cronbach's α Coefficient	Composite reliability	Average Variance Extracted
Willingness to share	PHID1	0.873	0.837	0.902	0.855
	PHID2	0.870			
	PHID3	0.861			
Attitude	A1	0.912	0.943	0.959	0.873
	A2	0.944			
	A3	0.935			
	A4	0.907			
Perceptual Behavioral Control	PBC1	0.844	0.856	0.913	0.868
	PBC2	0.902			
	PBC3	0.898			
Social Rewards	SR1	0.873	0.827	0.897	0.800
	SR2	0.808			
	SR3	0.905			
Past Positive Experiences	PPE1	0.874	0.875	0.923	0.778
	PPE2	0.926			
	PPE3	0.882			
Disease Severity	DS1	0.982	0.876	0.932	0.745
	DS2	0.884			
Information sensitivity	IS1	0.942	0.924	0.952	0.754
	IS2	0.914			
	IS3	0.939			

Table E.2 Table of correlation coefficients

Construction	Attitude	Disease severity	Information sensitivity	Perceptual Behavioral Control	Willingness to share	Past Positive Experiences	Social Rewards
Attitude	**0.855**						
Disease Severity	0.150	**0.873**					
Information Sensitivity	-0.256	0.161	**0.868**				
Perceptual Behavioral Control	0.668	0.149	-0.168	**0.778**			
Willingness to share	0.812	0.103	-0.316	0.584	**0.754**		

Past Positive Experiences	0.744	0.072	-0.044	0.609	0.614	**0.800**	
Social Rewards	0.633	0.011	-0/058	0.621	0.626	0.709	**0.745**

Note: The diagonal data are the arithmetic square root of the corresponding constructed AVE

Table E.3 Table of Cross Factor Loadings

Construction	Attitude	Disease severity	Information sensitivity	Past positive experience	Perceived behavior control	Social reward	Willingness to share
**A1**	**0.912**	0.139	-0.256	0.606	0.598	0.575	0.701
**A2**	**0.944**	0.156	-0.183	0.712	0.638	0.616	0.7
**A3**	**0.935**	0.144	-0.27	0.678	0.631	0.586	0.728
**A4**	**0.907**	0.114	-0.237	0.615	0.605	0.565	0.618
**DS1**	0.173	**0.982**	0.12	0.091	0.145	0.022	0.133
**DS2**	0.07	**0.884**	0.234	0.014	0.135	-0.018	0.014
**IS1**	-0.235	0.132	**0.942**	-0.031	-0.168	-0.048	-0.286
**IS2**	-0.253	0.225	**0.914**	-0.085	-0.135	-0.075	-0.302
**IS3**	-0.227	0.091	**0.939**	-0.006	-0.168	-0.039	-0.294
**PBC1**	0.586	0.187	-0.24	0.457	**0.844**	0.479	0.549
**PBC2**	0.604	0.106	-0.131	0.565	**0.902**	0.564	0.472
**PBC3**	0.579	0.103	-0.079	0.585	**0.898**	0.597	0.522
**PHID1**	0.663	0.133	-0.245	0.512	0.475	0.545	**0.873**
**PHID2**	0.639	0.114	-0.281	0.524	0.534	0.545	**0.87**
**PHID3**	0.638	0.022	-0.296	0.565	0.511	0.54	**0.861**
**PPE1**	0.618	0.085	0.009	**0.874**	0.541	0.634	0.547
**PPE2**	0.633	0.055	0.008	**0.926**	0.543	0.615	0.517
**PPE3**	0.647	0.054	-0.131	**0.882**	0.548	0.654	0.582
**SR1**	0.541	-0.034	-0.038	0.573	0.543	**0.873**	0.53
**SR2**	0.505	0.042	-0.185	0.567	0.491	**0.808**	0.541
**SR3**	0.591	0.019	0.059	0.689	0.571	**0.905**	0.55

Appendix F

Table F. Base Model Validation Results

Hypothesis	Paths	Coefficient	T test value	Significance	Variance explained R2
H1	Attitude —> Willingness to share	0.542	6.526	***	0.611
H2	Perceived Behavioral Control —> Willingness to share	0.078	0.957	NS	
H3	Social Rewards —> Willingness to share	0.214	2.254	**	
Control variables	Gender—> Willingness to share	-0.082	0.807	NS	
	Age—> Willingness to share	0.101	2.302	**	
	Educational level —> Willingness to share	-0.002	0.043	NS	
	Computer usage —> Willingness to share	0.039	0.776	NS	
H4	Past Positive Experiences —> Social Rewards	0.709	20.747	***	0.503
H5	Past Positive Experiences —> Attitude	0.687	16.310	***	0.571
H7	Disease severity —> Attitude	0.141	2.603	***	
H8	Information sensitivity —> Attitude	-0.248	5.740	***	
H6	Past Positive Experiences —> Perceived Behavioral Control	0.602	12.121	***	0.390
H9	Information sensitivity —> Perceived Behavior Control	-0.142	2.890	NS	

Note: *** represents significance at the 0.01 confidence level, ** represents significance at the 0.05 confidence level, * represents significance at the 0.1 confidence level, NS represents not significance
